# Occurrence of Pre- and Postoperative Stress Urinary Incontinence in 105 Patients Who Underwent Tension-Free Vaginal Mesh Surgery for Pelvic Organ Prolapse: A Retrospective Study

**DOI:** 10.1155/2014/643495

**Published:** 2014-02-06

**Authors:** Haruhiko Kanasaki, Aki Oride, Tomomi Mitsuo, Kohji Miyazaki

**Affiliations:** Department of Obstetrics and Gynecology, Shimane University, School of Medicine, 89-1 Enya-cho, Izumo, Shimane 693-8501, Japan

## Abstract

*Objective*. To examine retrospectively the occurrence of stress urinary incontinence (SUI) in patients who underwent transvaginal mesh repair (TVM) for pelvic organ prolapse (POP). *Methods*. The presence of preoperative SUI and postoperative changes in SUI was retrospectively analyzed for 105 patients who underwent TVM for POP between September 2009 and September 2012. *Results*. Preoperative SUI was observed in almost half of the patients (*n* = 50) who underwent TVM surgery. No significant differences were seen in patient age, pelvic organ prolapse quantification (POP-Q) stage, or primary POP complaint between those with and without preoperative SUI. Of the 50 patients with preoperative SUI, SUI was resolved in 14 (28%) following TVM surgery. Of the 55 patients without preoperative SUI, de novo postoperative SUI appeared in 26 (47.3%), of whom approximately half experienced resolution or improvement of SUI within 6 months postoperatively. There was no relationship between preoperative residual urine volume and occurrence of postoperative SUI. *Conclusion*. TVM surgery is a useful surgical method that can replace traditional methods for treating POP, but sufficient informed consent with regards to the onset of postoperative SUI is required.

## 1. Introduction

Japan already has a super-aging society, where 1 in every 5 people is aged 65 years or older. In such an aging population, the issue of pelvic organ prolapse (POP) is an increasing concern. Japanese gynecologists have traditionally performed curative surgical procedures such as vaginal hysterectomy (VH), anterior and posterior colpoplasty, and circumferential suture of the levator ani muscles for POP and especially for uterine prolapse. However, following reports by a French group of gynecologists of a tension-free vaginal mesh (TVM) surgery for the repair of POP using mesh without hysterectomy that has a favorable cure rate and low frequency of complications [1, 2], many Japanese gynecologists have switched to using this technique. In certain hospitals, this surgical procedure has replaced VH as the first-line surgical option for POP.

POP is attributed to vulnerability of the pelvic floor and often adversely affects quality of life and sexual function [3, 4]. It can also distort the lower urinary tract, resulting in stress urinary incontinence (SUI) and other voiding dysfunctions [5]. The aim of treatment for POP should, therefore, not only aim at anatomical restoration but also at maintaining or improving patients' quality of life.

As many POP patients present with lower urinary symptoms, it is vital that voiding function is evaluated preoperatively. It should also be evaluated postoperatively on followup because several studies have demonstrated that applying transvaginal mesh for anterior vaginal prolapse increases the risk of de novo SUI [6, 7]. In addition, other complications such as mesh erosion, de novo dyspareunia, and recurrent prolapse have been reported in women who have undergone TVM [8].

At the Department of Obstetrics and Gynecology, Shimane University School of Medicine, we have employed TVM surgery as the first-line surgical treatment for POP. We report here the clinical outcome of 105 patients with POP who underwent TVM surgery, focusing on the occurrence of pre- and postoperative SUI and examining the correlation between pelvic organ prolapse quantification (POP-Q) stage, predominant prolapsed organ, and residual urine volume.

## 2. Materials and Methods

Between September 2009 and September 2012, 105 women with POP underwent the TVM procedure at our department. Mean age at surgery was 69.4 ± 8.20 years; 15 women were ≤59 years, 37 were 60–69 years, 41 were 70–79 years, and 12 were ≥80 years. Those women with POP-Q stage ≥2 who provided written informed consent were scheduled for TVM surgery.

The presence of preoperative SUI was confirmed at the time of surgery and patients were designated into two groups: with and without preoperative SUI. In 48 patients, residual urine volume was measured once after voiding on the day prior to surgery.

The TVM surgical technique has been described previously [9]. Briefly, a monofilament polypropylene mesh (25 × 25 cm, Gynemesh PS; Ethicon, Somerville, NJ) is cut into a similar shape as that used in the Prolift system (Ethicon, Somerville, NJ). The procedure for the A-TVM begins with an anterior colpotomy. A full-thickness incision of the vaginal wall is made following sufficient fluid separation with epinephrine diluted 4 × 10^5^-fold. Cystocele correction warrants bilateral transobturator passage of the mesh to suspend it. The mesh arm on each side is passed into the paravesical region using a modified Emmet needle. The anterior subvesical strap into the arcus tendineus fasciae pelvis and the posterior subvesical strap is inserted into the arcus tendineus 1 cm from the sciatic spine. For the P-TVM to correct rectocele and/or uterine prolapse, a posterior colpotomy is performed longitudinally, and the mesh is placed under the vaginal wall. On each side, the mesh strap is passed into the pararectal space through the sacrosciatic ligament to become exteriorized in incisions located outside and below the anus. For total TVM (T-TVM) in patients with vaginal stump prolapse following hysterectomy, a one-piece prosthetic mesh consisting of two parts connected to each other is inserted into the anterior and posterior walls. The mesh is precut and adjusted according to the type of correction required. Traction over the exteriorized arms of the sling ensures correct positioning. After cystoscopy and digital examination of the rectum, the colpotomy is closed with a 2-0 PDS running suture without additional colpectomy.

Patients were discharged 3 days after surgery and monitored for postoperative complications on an outpatient basis at 1, 3, 6, and 12 months postoperatively. Postoperative SUI was defined as even one episode of a period of increased intra-abdominal pressure that SUI patients reported at the 1- and 3-month followups. Those patients without preoperative SUI who reported experiencing an episode of SUI after TVM for the first time were designated as having de novo postoperative SUI.

For analysis of POP-Q stage by age distribution, patients were divided into 4 groups: <60 years, 60–69 years, 70–79 years, and ≥80 years. For the analysis of predominant prolapsed organ by age distribution, we divided the predominant prolapsed organ into cystocele, uterine prolapse, rectocele, and vaginal vault prolapse.

Statistical analysis was performed using the chi-square test with *P* < 0.05 taken to indicate statistical significance.

## 3. Results

The characteristics of the 105 patients who underwent TVM surgery between September 2009 and September 2012 are shown in Table 1. Stage III was most commonly diagnosed POP-Q stage (*n* = 73), followed by stage II (*n* = 23) and stage IV (*n* = 9). No significant difference in the degree of POP progression was found for age distribution.

In regard to the predominant prolapsed organ, the most common was cystocele in 63 patients (60%), uterine prolapse in 29 (27.6%), vault prolapse in 7 (6.7%), and rectocele in 6 (5.7%). No significant difference was found between the POP primary lesion and age distribution, as shown in Table 2.

Almost half of the 105 patients who underwent TVM surgery had preoperative SUI (*n* = 50). No significant difference in the frequency of preoperative SUI was seen between the age groups. In addition, irrespectively of POP-Q stage or predominant prolapsed organ, there was no significant relationship observed with the presence of preoperative SUI (Table 3).

Analysis of the occurrence of postoperative SUI revealed that from the 55 patients who did not have preoperative SUI, 28 (50.9%) did not have SUI postoperatively; the remaining 26 (47.3%) had de novo postoperative SUI. No differences were observed in the postoperative SUI incidence rate due to differences in the predominant prolapsed organ or when investigated by POP-Q classification (data not shown). Of the 50 patients with preoperative SUI, 14 (28.0%) reported no postoperative SUI and the remaining 35 (70%) observed the same degree of SUI as they had preoperatively (Table 4).

From among the 26 patients with de novo postoperative SUI, 23 attended followup for ≥6 months. Of these 23 patients, 5 (21.7%) reported the resolution of SUI and 8 (34.7%) reported that symptoms were milder despite still being present. These 13 patients who experienced resolution or alleviation comprised half of those in whom de novo SUI appeared postoperatively following TVM. Anticholinergics were prescribed for the 4 patients whose symptoms did not improve, and of these, 1 patient underwent incontinence surgery using tension-free vaginal tape (TVT surgery) (Table 5). When the 35 patients with preoperative SUI and in whom SUI persisted after TVM were asked about the course of their symptoms at the 3-month followup, 25 (71.4%) reported that SUI was the same as preoperatively and 10 patients (28.6%) reported that it had worsened. Of these, 4 patients (11.4%) were prescribed medication (Table 6).

Residual urine volume measurements taken in 48 patients prior to TVM surgery were investigated with relation to SUI. When the number of patients with a preoperative residual urine volume ≥50 mL and those with volume <50 mL was compared according to POP-Q stage, no relationship was observed between them. There was also no relationship observed between the presence of preoperative SUI and preoperative residual urine volume (Table 7) or between preoperative residual urine volume and the presence of postoperative SUI (Table 8).

## 4. Discussion

De novo SUI is one of the complications of POP surgery in patients without preoperative SUI [10, 11]. In the present study, patients who underwent POP surgery at Shimane University Hospital in the past 3 years were studied retrospectively to investigate the relationship between SUI and POP. Firstly, the POP-Q stages of 105 patients who underwent TVM surgery were classified by age, but there were no significant differences in terms of the degree of POP progression and age distribution. In addition, even when POP was classified according to a report by DeLancey, where injury at Stage I leads to uterine and vault prolapse, and injury at Stage II leads to cystocele and rectocele formation [12], no difference was observed between the age groups when predominant prolapsed organ was compared. These results do not show that POP itself progresses as age increases but rather that there are no differences between the age groups when the main site of injury is compared.

Pelvic organ prolapse not only consists of the symptoms of organ descent but also causes a high rate of lower urinary tract symptoms, including difficulty in voiding, dysuria, and incontinence [13]. In the present study, the presence of preoperative SUI in 105 patients who underwent mesh surgery was investigated. There was no difference in the presence of SUI between patients with and without preoperative SUI, regardless of whether the presence of SUI was investigated by age, POP-Q stage, or by predominant prolapsed organ. This is consistent with a report by Araki et al. [14] stating that the detrusor muscle function is widely distributed in POP patients.

Postoperative SUI is a well-known complication of POP surgery, but even in our study, 47.3% of the 55 patients without preoperative SUI experienced de novo postoperative SUI. This implies that de novo SUI is a result of the resolution of urethral obstruction by anatomical reconstruction. Therefore, the fact that approximately half of the patients without preoperative SUI will develop it postoperatively needs to be the subject of informed consent. Meanwhile, although a third of the 50 patients with preoperative SUI reported resolution postoperatively, 70% still had symptoms of SUI. TVM surgery did improve SUI in some cases, but even still, we suggest it would be better for patients if their expectations regarding SUI improvement by TVM surgery are not too high. After TVM surgery, approximately half of the patients developed de novo SUI, but of those approximately half reported that SUI was resolved within 6 months or it had subsided to the extent that did no longer bothered them. Conversely, for 17%, it became a problem that affected their everyday lives and medication was prescribed; incontinence surgery was performed for only one patient (4.3%). Even if postoperative SUI appears only transiently, a small number of serious cases may require surgery. Thus, even if postoperative SUI appeared only transiently in our patients, we considered whether or not surgical treatment should be expedited, keeping in mind the possibility of relieving the symptoms.

No association was observed between the preoperative residual urine volume and presence of preoperative SUI, so predicting the onset of SUI postoperatively is considered difficult.

As stated earlier, gynecologists should routinely make a detailed evaluation of voiding function as a basic minimum when POP surgery is scheduled. It has previously been suggested that preoperative evaluation of detrusor function or urethral function might help predict urinary outcome in patients with POP [14, 15]. Since TVM is associated with the risk of inducing SUI, a condition not usually treated by obstetricians and gynecologists, such professionals should improve their understanding of the pathogenesis of various types of urinary incontinence and specific tests, such as urodynamic study, as well as cooperating with urologists in treating POP. As the number of obstetricians and gynecologists adopting TVM for the treatment of POP is expected to increase, surgeons should know that sufficient informed consent with regards to the onset of postoperative SUI is required.

## 5. Conclusion

TVM surgery is a useful surgical method that can replace traditional methods for treating POP, but sufficient informed consent with regards to the onset of postoperative SUI is required.

## Figures and Tables

**Table 1:** The characteristics of the 105 patients who underwent TVM surgery.

POP-Q stage	Patient age				Total patients
	<60 years	60–69 years	70–79 years	≥80 years	
Stage II	4	5	11	3	23
Stage III	10	29	27	7	73
Stage IV	1	3	3	2	9

Total	15	37	41	12	105

No significant difference with *m* × n  *χ* ^2^ test.

**Table 2:** Predominant prolapsed organ and age distribution.

	Cystocele	Uterine prolapse	Rectocele	Vaginal vault prolapse	Total
<60 years	12	2	0	1	15
60–69 years	25	11	0	1	37
70–79 years	19	12	6	4	41
≥80 years	7	4	0	1	12

Total	63	29	6	7	105
Percentage (%)	60.0%	27.6%	5.7%	6.7%	100%

No significant difference with *m* × *n* *χ* ^2^ test.

**Table 3:** Pre- and postoperative SUI in the patients who underwent TVM surgery.

	Without preoperative SUI (*n* = 55)	With preoperative SUI (*n* = 50)
Patient age		
<60 years	7	8
60–69 years	15	22
70–79 years	27	14
≥80 years	6	6
POP-Q stage		
Stage II	13	10
Stage III	35	38
Stage IV	7	2
Predominant prolapsed organ		
Uterine prolapse	18	11
Cystocele	30	33
Vaginal vault prolapse	2	5
Rectocele	5	1

No significant difference with *m* × *n* *χ* ^2^ test.

**Table 4:** Status of SUI after TVM surgery.

	Without postoperative SUI	With postoperative SUI	Unknown
Without preoperative SUI (*n* = 55)	28 (50.9%)	26 (47.3%)	1 (1.8%)
With preoperative SUI (*n* = 50)	14 (28.0%)	35 (70.0%)	1 (2.0%)

No significant difference with 2 × 2  *χ* ^2^ test.

**Table 5:** Outcome of patients with de novo postoperative SUI (*n* = 26).

Attended followup for >6 months	23	Percentage (%)
SUI resolved	5	21.7%
SUI lessened	8	34.8%
SUI unchanged	6	26.1%
SUI worsened	4	17.4%
(Prescribed medication)	4	17.4%
(TVT surgery)	1	4.3%

**Table 6:** Outcome of patients with both pre- and postoperative SUI (*n* = 35).

		Percentage
SUI roughly the same	25	71.4%
SUI worsened	10	28.6%
(Prescribed medication)	4	11.4%

**Table 7:** The relation with POP-Q stage and preoperative SUI with residual urine volume.

	Residual urine <50 mL	Residual urine ≥50 mL	Total
Stage II	9	3	12
Stage III	20	11	31
Stage IV	4	1	5
Total	**33**	**15**	**48**

Without preoperative SUI	19	8	27
With preoperative SUI	14	7	21
Total	**33**	**15**	**48**

No significant difference with m × n  *χ* ^2^ test.

**Table 8:** Residual urine volume with postoperative SUI.

		With postoperative SUI	Without postoperative SUI
Residual urine <50	33	20	13
Residual urine ≥50	15	8	7

No significant difference with 2 × 2  *χ* ^2^ test.
